# An ethnographic investigation of the maternity healthcare experience of immigrants in rural and urban Alberta, Canada

**DOI:** 10.1186/s12884-015-0773-z

**Published:** 2016-01-27

**Authors:** Gina M Higginbottom, Jalal Safipour, Sophie Yohani, Beverly O’Brien, Zubia Mumtaz, Patricia Paton, Yvonne Chiu, Rubina Barolia

**Affiliations:** Mary Seacole Professor of Ethnicity and Community Health School of Health Sciences, University of Nottingham, Rm 1976, A Floor, South Block Link Queen’s Medical Centre, Nottingham, NG7 2HA UK; University of Alberta, Alberta, Canada; Department of Health and Caring Sciences, Linnaeus University, Building: K2244, 35195 Vaxjo, Linnaeus, Sweden; Department of Educational Psychology, University of Alberta, 6-107D Education North, Edmonton, Canada; Faculty of Nursing, University of Alberta, 3rd Floor Edmonton Clinic Health Academy, 11405 87th Avenue, Edmonton, T6G 1C9 Canada; School of Publin Health, University of Alberta, 3rd Floor Edmonton Clinic Health Academy, 11405 87th Avenue, Alberta, T6G 1C9 Canada; Alberta Health Services, College and Association of Registered Nurses of Alberta, 11620 168 Street, Edmonton, T5M 4A6 Canada; Multicultural Health Brokers Coop, # 301, 9955-114 Street, Edmonton, T5K 1P7 Canada; School of Nursing and Midwifery, Aga Khan University, stadium Road, Karachi, 74800 Pakistan

**Keywords:** Diversity, Ethnographic study, Healthcare access, Maternity care, Immigrant woman, Canada

## Abstract

**Background:**

Canada is among the top immigrant-receiving nations in the world. Immigrant populations may face structural and individual barriers in the access to and navigation of healthcare services in a new country. The aims of the study were to (1) generate new understanding of the processes that perpetuate immigrant disadvantages in maternity healthcare, and (2) devise potential interventions that might improve maternity experiences and outcomes for immigrant women in Canada.

**Methods:**

The study utilized a qualitative research approach that focused on ethnographic research design and data analysis contextualized within theories of organizational behaviour and critical realism. Data were collected over 2.5 years using focus groups and in-depth semistructured interviews with immigrant women (n = 34), healthcare providers (n = 29), and social service providers (n = 23) in a Canadian province. Purposive samples of each subgroup were generated, and recruitment and data collection – including interpretation and verification of translations – were facilitated through the hiring of community researchers and collaborations with key informants.

**Results:**

The findings indicate that (a) communication difficulties, (b) lack of information, (c) lack of social support (isolation), (d) cultural beliefs, e) inadequate healthcare services, and (f) cost of medicine/services represent potential barriers to the access to and navigation of maternity services by immigrant women in Canada. Having successfully accessed and navigated services, immigrant women often face additional challenges that influence their level of satisfaction and quality of care, such as lack of understanding of the informed consent process, lack of regard by professionals for confidential patient information, short consultation times, short hospital stays, perceived discrimination/stereotyping, and culture shock.

**Conclusions:**

Although health service organizations and policies strive for universality and equality in service provision, personal and organizational barriers can limit care access, adequacy, and acceptability for immigrant women. A holistic healthcare approach must include health informational packages available in different languages/media. Health care professionals who care for diverse populations must be provided with training in cultural competence, and monitoring and evaluation programs to ameliorate personal and systemic discrimination.

## Background

International migration, largely from east to west and south to north, results in increasing ethnocultural diversity in immigrant-receiving nation states [1]. Canada is among the top immigrant-receiving countries globally [2]. According to Statistics Canada, 20 % of the total population increase in the last decade (approximately 2,000,) consists of individuals born outside Canada [3]. This proportion was also seen in rural areas [4]. A gender analysis demonstrates that almost 50 % of immigrants are women [5], many of whom are in the reproductive phases of their lives. Research evidence indicates that immigrant women experience many challenges in receiving both community and hospital maternity care [6–8]. Canadian health policy is committed to providing safe, accessible, and equitable care for mothers and their children [9, 10]. This goal is congruent with the G8 Millennium Goals. This study explores critical points and barriers faced by immigrant women who seek maternity care. Our target population was heterogeneous and may have included economic migrants and skilled workers, temporary foreign workers, documented and undocumented residents, refugee claimants, refugees, asylum seekers, and students.

### Maternity care in Canada

With the exception of the Aboriginal population, maternity care in Canada is a provincial matter and public health insurance plans cover most expenses related to childbirth [11]. Physician specialists provide maternity services, and almost half of family doctors (47 %) include prenatal care as part of their practice [11, 12]. Standard practice is for pregnant women to receive prenatal care every 4 to 6 weeks in the early stages of pregnancy and every 1 to 2 weeks in the third trimester [11]. Midwives also provide a full scope of maternity care for about 3 % of women in Canada. Hospital stays have dramatically shortened since 1980 with current lengths of maternity stay being an average of one to two days. Community health services provide prenatal classes and postpartum visits by public health nurses (typically a phone call and home visit within 24 and 48 hours, respectively, of hospital discharge). The overriding goal of the latter is to assess and prevent postpartum problems and to enhance maternal and newborn wellbeing and adjustment [13].

### Why focus on maternity care?

Access to healthcare, including maternity care, and the quality of services received by immigrants are influenced by low health literacy levels [14], stigma and mental health problems [15], language barriers, cultural barriers, lack of provider cultural competence, and lack of social support and isolation [16–18], despite policy suggesting that care is to be provided equally for all members of society [19, 20]. Maternity care that is adapted poorly to serve diverse cultural populations can result in low birth weight and preterm birth, which can impact the short or long-term health of women and children [12, 19, 21, 22]. Inadequate health assessment and communication issues between care givers and their clients may encourage unnecessary interventions, thereby wasting precious resources [23]. More information about the diversity of experience and the factors that shape the experiences of immigrant women in the Canadian healthcare system will help us to improve maternity health in Canada [24, 25].

### Theoretical framework

Immigrant women’s access to and experiences within the healthcare system may be viewed usefully within the context of both organizational behaviour and critical realism theories. Organizational behaviour theory concerns the study of individuals’ interactions within specific units of an organization [26] in which there is structure to meet specific goals for certain populations [27]. Within this theory there is no universal or ideal way to manage organizations; the organization is thought to require flexibility and plasticity based on circumstances and environmental factors [28]. A change in demographics, such as an increase in diversity, will impact the healthcare system, and system adjustment will be required based on the emerging needs of the precursor of demographic change. [29]. A major factor is the level of cultural competence of healthcare providers and at a macro level, the organizational cultural competence. Many definitions of cultural competence prevail in the literature. We found the following pertinent for our use: within the healthcare sector is “*the ability of healthcare providers and healthcare organizations to understand and respond effectively to the cultural and linguistic needs brought by patients to the healthcare encounter*” [30]. As an overall aim is to have the ongoing capacity to provide equitable healthcare services for diverse populations [31], healthcare organizations need to continually make the changes and adjustments that will provide culturally appropriate care for these populations [29]. More specifically, the organization needs to provide timely, efficient, safe, and equitable care for all clients regardless of gender, age, ethnicity, and socioeconomic status [26, 32]. In this study we considered the *maternity healthcare setting* in Canada, and investigated the experiences of immigrant women regarding access to and navigation of Canadian maternity services.

Critical realism theory within the social sciences provides analysis of the nature of the social world by incorporating two main features: (a) understandings of the dialectical interplay between social structures and human agency, in this case maternity care services and immigrant women, and (b providing critique of the prevailing social order, which for this study is the notion that immigrant women may be regarded as “the other” [33–35]. In healthcare science, critical realism can help the researcher to understand and explain health related phenomenon [36, 37]. Critical realism theory applies to investigations of complex and multilayered contexts; an example of such a context is the healthcare service, in which therapeutic encounters occur and dialogical interaction exists between patients, health professionals, managers, and policy makers [35, 37]. Healthcare practitioners may have different ethnocultural and socioeconomic backgrounds that influence their practice and subsequently affect care outcomes [36]. Critical realism is applied in this study to accommodate the existence of multiple social perspectives (immigrant women patients, healthcare and social service providers, and other key stakeholders), which may or may not coalesce. It also enables us to identify existing barriers at both macro and micro levels, taking account of the notions of personal agency and healthcare structure [34, 35, 37].

## Methods

**Fig. 1:**
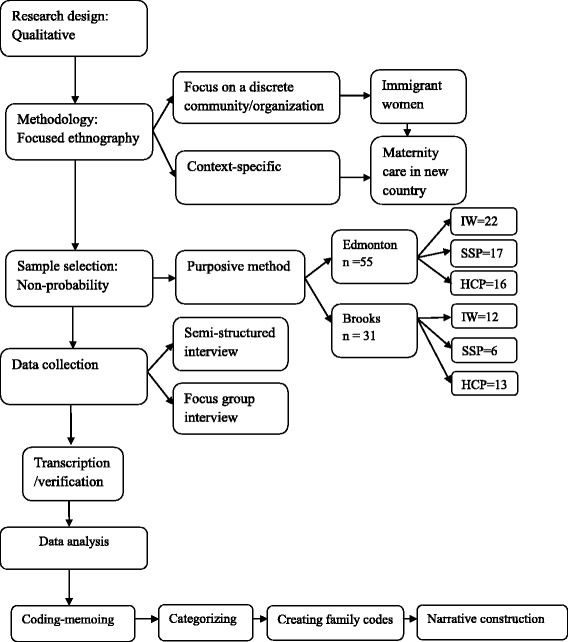
Qualitative research design of the study (IW = immigrant woman/women; HCP = healthcare provider; SSP = social service provider/other stakeholder)

This qualitative, focused ethnographic study was part of larger mixed- methodological research project conducted to (1) generate new understandings of processes that perpetuate disadvantages to immigrant women who seek maternity services, (2) provide generic theoretical and practical suggestions of how health systems can promote better outcomes for immigrants. In the present study, we investigated *barriers to accessing* maternity healthcare services by immigrant women by applying a focused ethnographic methodological approach deemed suitable to elicit the complexities within both *access* to services and the *experiences* of immigrant women [38–40]. A focused ethnography (Fig. 1) was employed because of (1) our focus on a discrete community (immigrant women) and setting/organization (maternity healthcare system), and (2) the problem-focused and context-specific nature of the phenomenon of accessing maternity care in a new environment [41–44]. As Muecke [42] and Fetterman [39] clarified, there is a distinction between focused ethnography and traditional anthropologic ethnographies. Traditional anthropological approaches require the researcher to remain within the given society for sustained periods in order to facilitate cultural immersion.

### Study settings

The study locations included a metropolitan city and a smaller, rural town in a Canadian province. The study sites were selected to provide insights into immigrant experiences in both rural and urban locations. The provincial location is a popular destination for immigrants to Canada, and in 2011 the urban site ranked fifth for its number of new immigrants [45]. Canada Census 2006 documented one million people, of which 189,775 had an immigrant background and most of these (174,729) belonged to a visible minority group [46]. There is considerable heterogeneity within immigrant communities [47] and the study site receives refugees from Sudan, Afghanistan, Somalia, Democratic Republic of Congo, Sierra Leone, Liberia, Colombia, Iran, Iraq, and other countries. In the rural town location, 2,135 people (17 %) were identified as visible minorities and 16.6 % as immigrants [4]. In 2006, almost 50 % of those identifying as visible minorities in the entire health region resided in the rural town of our study. The top source countries for refugees arriving in Canada over the last few years have been Ethiopia, Sudan, Somalia, Burundi, Democratic Republic of Congo, Afghanistan, Pakistan, and China; 75–80 % of the immigrants have either basic or no fluency in English.

### Sample and recruitment

Focused ethnographers aim to investigate a particular phenomenon in a specific setting and/or for a specific group of people [41], thus we generated a nonprobability purposive sample who could inform us about the experiences of immigrant women in maternity care within their area (Fig. 1). Those eligible for inclusion were (1) immigrant women with current or recent (previous 2 years) experience of using maternity services while residing in either the urban or rural location, (2) healthcare providers having experience with providing perinatal care to immigrant women, and (3) stakeholders, including social service providers and decision makers, who have a mandate or involvement in immigrant women’s health or service provision. The study team worked closely with an immigrant-serving agency, the Multicultural Health Brokers Co-operative (MHBC), where cultural health brokers (employed as community researchers) assisted with both recruitment of immigrant women and language interpretation during interviews. The MHBC offers liaison, referral, and information services, often through trained cultural health brokers, to help immigrants and refugees access and navigate the health system. The brokers informed the immigrant women of the study using a prepared information package, which was translated if requested. The bi-lingual brokers also gained initial consent from the women for the research team to contact them to arrange procedures for obtaining informed consent and data collection. In the rural location, it was routine for participating healthcare or social service providers to assist with informing and gaining contact consent from immigrant women in their care. Snowball sampling was used on several occasions, whereby participants would pass along the research team contact information to suitable acquaintances or colleague.

### Data collection

We used semistructured individual and focus group interviews to obtain data. Interviewers were guided by an interview topic guide having topic prompts which shifted in focus from an immigrant’s general impression of Canadian maternity services (including comparison to the home country) to more specific topics relevant to specific experiences of the immigrant [48], including barriers and facilitators that affected access to and reception of care and impressions of care provision. The interviews were audiotaped (with permission), transcribed verbatim by a professional transcriptionist, and then verified by the interviewer. Data from the focus groups were also verified by the cultural health broker who served as the interpreter for the immigrant women. Data were gathered over a two year period and recruitment ceased once data saturation was reached, as confirmed through our iterative data analysis process. All data were collected by female researchers trained in qualitative interviewing to prevent difficulties for immigrant women in expression or sharing of maternity experiences. Although our recruitment efforts facilitated recruitment of immigrant women of all ethnocultural groups, there is always the possibility that our results have some inherent bias from failure to capture differing perspectives.

### Participant characteristics

The background of immigrant women (IW) participants was diverse, with 12 from Sudan, 8 from the Philippines, 6 from China, 2 from Columbia, and one each from Tajikistan, India, Mauritania, Pakistan, and Eritrea. Eighteen (78.3 %) of the social service providers/stakeholders (SSP) and 8 (27.6 %) of the healthcare providers (HCP) had immigrant backgrounds. Seven (24.1 %) among the HCP sample were bilingual whereas 96.6 % of the SSP were bilingual; 73.5 % of the IW reported that they could understand English. The average age of the IW sample was 35.5 years and the majority of the HCP and SSP participants were female.

### Data analysis

Principal investigators, coinvestigators, collaborators, and trainees reviewed transcripts to gain preliminary interpretations. Several reflective team analytical meetings were scheduled to provide and train novice team members in collaborative, reflexive analysis and interpretation. Drawing upon the tenets of critical realism we sought to establish both individual and structural factors for the existence of the phenomenon focusing on the stratification of the women’s experiences in respect of a) the empirical b) the actual or observed c) the real. Data were analyzed with the aid of Atlas.ti (ATLAS.ti Scientific Software Development GmbH, Germany) [49] following Roper and Shapira’s [40] framework for analysis of ethnographic data. Data were first coded with descriptive labels, then categorized into “code families” based on identified patterns. During this process, analytical and theoretical memos were written for narrative construction of the results (Fig. 1).

### Ethical approval

Ethical approval was granted for this study by the institutional review board at the University of Alberta, Edmonton, Alberta, Canada, that is, the Health Review Ethics Board – Panel B.

Written informed consent to participate was obtained from all participants after emphasizing the participant’s right to withdraw at any time. Participants were informed of the study using both written and verbal means; interpreters were present when necessary throughout the consent and interview procedures. All information collected from this study is being kept secure; only individuals designated as research personnel have access to the data.

## Results

A total of 86 individuals participated in this study (Table 1). The group of healthcare providers included nurses (n = 16), midwives (n = 2), a social worker (n = 1), a dental assistant (n = 1), family physicians (n = 7), and obstetricians (n = 2). The social service providers/stakeholders included commissioners of health services, provincial politicians, and representatives of immigrant support agencies. All data from the rural location were gathered using semistructured individual interviews (SSI); in the urban setting, three focus group interviews (FGI) were also used, two with immigrant women (n = 12) and one with social service providers (n = 12).

**Table 1:** Demographics of the participating immigrant women (IW), social service providers (SSP; this includes some other key stakeholders), and healthcare providers (HCP)

Items	IW	SSP	HCP
N (%)	n = 34 (39.5 %)	n = 23 (26.8 %)	n = 29 (33.8 %)
Rural Town, n = 31 (36.5 %)	12 (13.9)	6 (7.0)	13 (15.1)
Urban Town, n = 55 (63.5 %)	22 (25.6)	17 (19.8)	16 (18.6)
Sex/female	34 (100)	22 (95.6)	27 (93.1)
Age/mean (SD)	35.47 (5.56)	45.23 (11.56)	46.03 (10.21)
Immigrant (Yes)	34 (100.0)	18 (78.3)	8 (27.1)

**Fig. 2:**
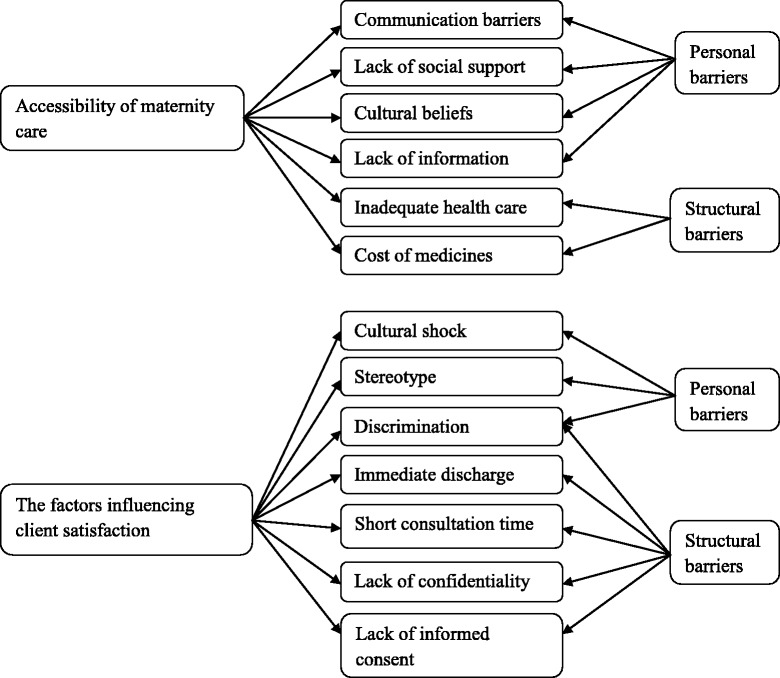
Summary of findings grouped by major themes (left side) and categorized by whether they represent personal and structural barriers (right side) to access and navigation of maternity services by immigrant women.

Data analysis focused on answering the specified research questions. Participant answers were referred to their stated personal narratives and to structural factors mentioned that would limit the accessibility to maternity services by immigrant women (Fig. 2). Two major themes were identified that affected the maternity care experiences of immigrant women in this study: (a) *accessibility of maternity care* and (b) *factors influencing client satisfaction*.

### Accessibility of maternity services

Although maternity care is available for all women in Canada regardless of their ethnicity, age, religious beliefs, and/or immigration background, several issues were found to be limiting factors for IW. Communication barriers, lack of information, lack of social support and isolation, cultural beliefs, inadequate healthcare services, and cost of medicines/services were all identified as potential risk factors for not accessing maternity services in a timely manner.

#### Communication barriers

A limited competence in the English language was considered a “communication barrier.” Although there are two official languages in Canada (English and French), only English is needed to enable effective communication in most areas of the western Canadian provinces. IW participants indicated that a communication barrier limited their opportunity to express their feelings and articulate their situation/problems, as well as to understand the many medical terms that arose through dialogue within the system. This problem was not addressed with comprehensive interpretation services. The predominant healthcare provider provided a telephone translation service, but this appeared to be underutilized. Consequently, IW having limited knowledge of English may face a major limitation in respect to the utilization of services. One woman in this situation stated:

…the nurse came to my house to visit me and she talk about lots of things but I didn’t understand … so whatever she asked me, I said, yeah, yeah, yeah. I just wanted to say, oh, you just leave. (IW-FGI-Urban Town).

In some cases, the women felt intimidated to the extent that they were afraid to ask questions:

… sometimes when we get into hospital … they speak very, like very fast English, and being new to the country you don’t – you are not really used to the accent, you know. And so, and they are scared to ask questions. (IW-FGI-Urban Town)

A HCP worried about whether clients understood everything:

It’s usually more on the gut feel that this person has no idea what you are talking about, no matter how slowly and simple you explain it. It’s just not getting through … I had someone send one of my patients for a triple screen which came back nicely with a positive for Down Syndrome and I couldn’t explain that in Arabic. (HCP-Rural Town)

Interestingly, the HCP failed to acknowledge that interpretation services might be required to render professional accountability and to ensure key healthcare information was conveyed.

Limited fluency in English sometimes prevented an IW from participating in prenatal classes; an IW FGI participant stated, “I didn’t quite understand because it’s in English and so I only attended I think two times and then I just quit because I didn’t quite understand.” To help overcome this problem in the healthcare sector, albeit mostly in tertiary care centres, professional interpreters or a language service (e.g., an online service based in the United States is currently under contract in the rural location) is made available. While these efforts reduce communication barriers, there seem to be challenges in regard to their being “time-consuming,” unavailable in delivery rooms and during the busy night hours, Alternatively, many immigrants bring along a family member (often their child) or a friend to assist with their communication, but this approach can lead to confidentiality and ethical issues.

#### Lack of information

Immigrant women may be familiar with the healthcare services in their home countries but unsure about how to navigate the system in Canada. Women may not use services or exercise their options if they are unaware of their existence. Participants in our study commonly articulated that limited language fluency was a major barrier to gaining information.

Many people, they are not informed, they don’t have information about any services…when you see something in English we are not interested to read it. (IW-Rural Town)

An HCP observed: “The thing is, when people come here, because they have no idea about community resources, they don’t go. They don’t come” (HCP-Rural Town).

This lack of information can result in an IW feeling “lost,” as explained by one immigrant FGI participant,

I was completely lost because I didn’t know … about the system and I had difficulties with the system again … that I did not understand initially there.

In some cases, it seems that there is too much written information, especially for an IW already overburdened with other issues, such as the immediate challenges of acculturation into a new environment. Furthermore, some immigrant women, especially those from more “oral” societies, prefer verbal transmission of information,

… I am also interested in them actually talking with you instead of just giving you the paperwork. (IW-Urban Town)

This participant clearly expressed a need for a greater level of therapeutic interaction and care from her healthcare provider.

#### Lack of social support and isolation

Many IW are used to receiving help and support during the pregnancy, birth, and the immediate postnatal period from their extended family in their home country, especially female relatives. Lack of social support can lead to a delay or irregularity of medical visits, thereby substantially affecting maternal physical and mental health.

Several of the IW commented on this topic, “I was very scared because my husband, he works at … and I have a baby, so I cannot go in Medicine Hat every like 15 days and month” (IW-Rural Town). Another IW pointed out: “I don’t have any. No neighbor, nobody here, no friend, no any family. This is too hard for me…Sometimes I sit only myself and I cry because I do not know what can I do” (IW-Rural Town). One IW mentioned that she was working in the late stage of her pregnancy since she was alone at home and wanted to be cared for at least by her colleagues, “I was in my eighth month I was still working. You know why? Because I don’t have anyone in the house. I was actually worried that I might give birth here and then I don’t have anybody.” Another issue related to social support is transportation. This is especially pertinent in a country that experiences adverse winter weather patterns (often with subzero temperatures extending to minus 40). For many IW arriving from more tropical environments, transportation and accessing services during winter can be a substantial challenge. Many of the women’s spouses worked long and/or irregular hours or did not have a driver’s licence. This was challenging given that spouses provided the primary support for the women participants in this study. Even women who had access to a vehicle and ability to drive thought that their pain or stress prevented them from going out alone. One SSP commented, “Some of them are pregnant and they are left alone by themselves, and transportation is a big issue for them, to go for the care.”

#### Ethnocultural beliefs and traditions

In some cases, a conflict existed between the values and beliefs in a woman’s native country and those in her new country. For example, several women viewed pregnancy as a natural life event and a physiological process during which they did not require special medical attention. The predominance of Western biomedicine and medicalisation of childbirth in Canada may be oppositional to these views. For example 98 % of all births in Canada are attended by a physician [50]. Both HCP and SSP commented on this topic, “They don’t believe that maternity care is [a] necessity … so to them pregnancy is not a disease, it’s like, I am not sick, I’m pregnant. It’s normal. I will get better” (SSP-Rural Town).

Health care providers in Canada tend to view pregnancy and childbirth as a “medical event” rather than a normal physiological process. Indeed this very issue may create “cultural dissonance.”

Another issue related to cultural values for some ethnocultural groups, particularly those of the Muslim religious faith, was the unacceptability of being cared for (especially being physically examined) by a male physician or nurse. This barrier seemed strong for some women and sometimes prevented them from using healthcare services: “They have request of not seeing male at all in their care. So that involves like the residents, so any medical students or resident who we have … that ethnic group refuse to have male” (HCP-Urban Town). Another HCP noted: “It’s been 35 years since they’ve had a pap because they don’t want to, come and see [a] male doctor” (HCP- Rural Town).

It was reported by one HCP that some IW may even reject the emergency services of a male obstetrician, “You know, an emergency during labour, in which case we needed the obstetricians to help but they didn’t want a man under no circumstances.” The comments elicited from care providers on this topic tended to be pejorative in nature rather than adopting a “woman centred” approach affording respect for religious and cultural diversity.

#### Inadequate healthcare system

Inadequacies in maternity care were largely related to a failure to provide culturally competent care and resulted in limited accessibility (to appropriate care) for IW. One identified barrier was related to finding a physician. In many cases, and apparently due to physician shortages, there was much difficulty in obtaining an appointment in a preferred clinic or family doctor’s office. Physicians were either not taking new clients or physicians were so busy that appointments were scheduled later in the antenatal period than desired. IW from both study sites commented: “So when I found out I was pregnant I need a family doctor first and then get a referral letter. So I called different doctors. They said, well, we don’t take new patients” (IW-Urban Town). An IW from the rural site noted:

It’s hard to find a family doctor because I phoned everyone … it took me for a while, and then you can’t go with him because his appointment [book] was full. I phoned a lot of clinics and then they cannot accommodate. (IW-Rural Town) It might take up to one year to find a family doctor. Moreover, after finally getting a family doctor and referral to a specialist clinic there was often another long waiting period because of the shortage of gynecologists or obstetricians. Some women complained that they received their first appointments in the advanced stages of their pregnancy. The issue of long wait periods was a great barrier to accessing care at an appropriate time.

#### Cost of medicines/services

The costs of prescribed medicine and in some instances services hindered the ability of some IW to receive appropriate healthcare. We did not collect data about healthcare insurance coverage, but it seems likely that these women did not have access to a comprehensive healthcare insurance. One IW described how the cost of one medication was prohibitively high for her, and a HCP provided specifics on some relevant drugs,

The doctor prescribed medication, which cost about a hundred dollars than she’s expected to pay for. Every month she’s supposed to pay to buy that … she doesn’t earn a lot of money and child tax is not a lot of money. (IW-Urban Town)

For example, one that we use for nausea and vomiting in pregnancy, Diclectin, is very expensive and it isn’t, if you don’t have a great plan it isn’t covered, or if you don’t have a plan it’s very, very expensive. (HP-Urban Town)

Women with permanent residency receive universal health care coverage, and would have issues only with drugs or services that were not covered. Healthcare insurance plans that pay for medications and services can be costly for individuals without an employer-paid program. Alternatively, immigrants or refugees who are covered under the federal government’s Interim Federal Health (IFH) plan may or may not get access to prescribed medication because of the large administrative burden to claim the costs [18]. This issue was brought up by one SSP in the Urban Town.

I have seen like some pharmacists, whereby when the family presented the, IFH, and they say: No, not covered … I do not deal with that. I know it’s covered but they don’t want to process it, and they make the client pay the money. (SSP-FGI- Urban Town)

One woman did not have enough money to pay for her operation,

At that moment we did not have the bucks to provide the $650, $600 dollars that (the operation) costs. Then I was going to do it, but that will be in the long term, because in that moment we did not have it. (IW-Urban Town)

### Factors influencing client satisfaction

IW who had experienced positive birth outcomes and the birth of healthy infants were very satisfied with their maternity care. Despite this, several important issues were identified that negatively influenced the IW’s perception of the quality of Canadian maternity care, they included: lack of understanding of the informed consent process, lack of respect for IW’s privacy, short consultation times, immediate discharge from the hospital after birth, discrimination and stereotyping, and cultural shock.

#### Full comprehension of the process of informed consent

A major finding was that gaining informed consent from many IW was challenging because the IW may not did not fully understand the informed consent process. HCPs often did not provide sufficient information, and used medical “jargon” to inform IW about consent, limiting comprehension. One HCP mentioned that when people are in pain, scared, or in a stressful situation they may not be “thinking right” and in these circumstances optimal  informed consent is not possible.

You’re trying to offer them an operation for what you feel are correct reason[s], so whether they understand English or don’t understand English, one could argue that their consent is perhaps not optimal in a stressful situation. (HCP- Urban Town)

This particular healthcare provider raises important concerns about the ethics of obtaining informed consent from individuals who do not appear to fully comprehend the informed consent concept.

An IW who thinks that a doctor will make decisions about her birthing method without consulting her will feel that her autonomy is compromised: They are going to take the baby with the C-section; I know that … there might be something that might cause the caesarean section. It can have side effect of, you know, progress of labour … if you have epidural you’re going to have a higher chance of C-section. (IW-FGI-Urban Town)

A woman said that she did not receive follow-up care since she said “No” to a question that she couldn’t understand:

Nurse asks me: you know about HPV test? So I said: I don’t know. She said to me: you suggest yes or no? So I said no because I can’t understand so that’s why I said no. (IW-Rural Town)

### Lack of privacy for a patient’s confidential information

Participants suggested that HCPs might fail to respect the privacy of patients by openly revealing confidential information.

I also have another client who has been hospitalized many times and people will come to visit her, and some people are curious and they will go to the front desk and ask: What happened to her? Why is she sick? And the nurse out loud told them. (HCP-FGI-Urban Town)

In such cases the ethical principle of confidentially has been transgressed.

#### Short consultation time

Some IW found the time for consultation with a physician or at clinics or hospitals was not long enough: “I was like, and scared and, because they are very fast. At my home place they’re ladies and they take more time if you want to talk or you want to tell something” (IW-Urban Town).

… [They are] quick [like]: “Okay, how are you feeling today?” “Oh, I feel better.”

“Oh. Okay. Do you feel painful?” “No.” “Okay, you can go.” Like I don’t care.

That’s the information but, I don’t care about you. Just the doctor in this way, just cold. (IW-Urban Town)

The preceding statement suggests a lack of trust and empathy during the health care interaction.

Another IW opined: “… this doctors have so little time for you anyway. They come five minutes maximum and then he’s gone, so he doesn’t ask you a lot of questions, if you forget, then next time” (IW-FGI-Urban Town).

“All I need [from physician] is to pay a little attention; more time and attention.

That is enough I think” (IW-Urban Town). This participant clearly expressed a need for a greater level of therapeutic care and enhanced communication.

#### Discharge immediately from the hospital

Some women felt that they were discharged too soon after their birth compared to policies (or past experiences) in their home country.

… in China after you deliver you can stay in hospital one week. And because in the first 72 hours [is] very important for the woman so they always stay in hospital and get some medicine to help … but here the doctor or the nurses just gave her the feeling that they want to push her out. (IW-Urban Town)

Although these women were satisfied with the quality of the care they received, they felt that HCPs were mostly concerned with “pushing” them out of the hospital: “After the C-section I was really weak in the hospital and I stayed there for three nights. Three nights and then you have to go home” (IW-Urban Town).

In another case, a woman who was not feeling well was discharged. In her home she fainted:

She preferred to stay in the hospital a little bit longer, maybe one more day or anything. But [the] OB didn’t care and sent her home and then she actually fainted or lost consciousness at home and then her husband would have to call 911 and send her back to the hospital. (IW-Urban Town)

#### Discrimination and stereotyping

The notion of cultural safety is predominant in Canadian health research, practice, and policy. Cultural safety is the recognition of power relationships between healthcare providers, patients, and clients [51, 52]. Recognition is required of the colonial and often racist historical antecedents that have created these power relationships, and the promotion of well-being [51, 53]. Key components of cultural safety are that we, as health professionals and researchers, are all bearers of the culture, that the unequal power relationships within society need to be recognized, and that the historical antecedents of these unequal power relationships need to be acknowledged [51]. Hence, the concept recognizes the processes of colonization that many immigrant women may have experienced in their home countries, and we acknowledge the primacy of white Eurocentric perspectives in the Canadian context. The concept has been widely embraced by Aboriginal groups in Canada, but the key tenets and axioms of cultural safety are equally pertinent to observe in research with immigrant populations [54, 55].

SSP and HCP participants reported some extent of discrimination and stereotyping based on one’s phenotype, color, origin, age, and body size.

I never thought that there was as much discrimination in Urban Town until I started working in here with more immigrant people … . (SSP -Urban Town) Probably it’s the people of colour versus the white. It may not be – because you don’t know if they’re citizen or not. Whether you’re a citizen or not, if you are white then there is a different kind of treatment, like more special treatment than, you know, other. (SSP-FGI-Urban Town)

Some participants thought that people are treated differently, based on dominant societal stereotypes, with respect to their specific ethnocultural practices. For example the tendency for immigrant women to have larger families than Canadian women: “We tend to have many like the East Africans, Somalia, Sudan, some Ethiopian, Eritrea. They tend to have kids every eleven months. They go up to eight, nine, ten. [laughs]” (HCP-Urban Town). Stereotyping based on ethnicity was also discussed by participants:

Most of the immigrant and mostly refugees, I think they come to Canada and everything to be given to them, so they treat us a little bit like a hotel … and some of them are very demanding … They are all quite easy except for the [ethnic group name] . They are the most demanding. (HCP-Rural Town).

It was reported that older pregnant women from African communities get targeted for more prenatal care, in the form of more medical tests, and even get “ear-marked” for

C-section birthing:

When you are pregnant and if you are over 30 …in the African community, the doctors scared to hell. You have to do this, you have to do that. You do this, we have to test this, we have to give you the amnio … so start talking to you about C-section. (IW-FGI-Urban Town)

You assess basically at the door and you know what’s happening and then you, you know, you plan your action before the patient hits the stretcher sort of … like I‘ve had practitioners say: you know what? All those people from such and such a country; they all have more than one wife. (HCP-Urban Town)

#### Cultural shock

Cultural differences and different approaches to maternity care were reported as one of the main factors influencing satisfaction with care received by IW in maternity healthcare settings [25, 56, 57]. Acculturation is a complex process and location to a new country inevitably means severing of social and family networks. The models of maternity care that women experience in their country of birth may differ widely from those which predominate in Canada. Without *culturally appropriate* healthcare delivery, however, a negative trajectory of events may occur that range from simple miscommunication to life-threatening incidents [58, 59]. The danger is especially severe during the perinatal period, which is for women and their children a vulnerable life stage and for immigrants a sensitive period of interaction with the Canadian healthcare system. Thus, cultural conflict may exist in respect of the differing models of care. For example, some IW perceived that the Canadian maternity care was more focused on prenatal and intrapartum periods than on the postpartum period, whereas in some ethnocultural groups more care is provided in the postpartum period:

The kind of treatment you get before you have a baby and then after you have a baby is completely different, and there’s things for us, we’re used to getting that treatment after we have birth, culturally … I think it’s the system, the ways it’s set up is that right after you have a baby it’s like: Get on it, like move on. Whereas in our culture we’re supposed to be looked after for 40 days, 60 days, so we expect that”

### Divergence in food choices and nutrition

Postmigration women may aspire to achieve acculturation in part by adopting a Western diet. For many immigrant women, however, Western diets and patterns of food consumption differ considerably from those in their country of origin. Evidence from this team’s ongoing research suggests that women may desire to adopt Western ways as a marker of acculturation, despite the fact that their original ethnoculturally-defined traditional diets and patterns of food consumption may be more desirable and healthy.In a recent research study that looked for ways to optimize maternity care for newcomer women, immigrant Sudanese women frequently requested pizza and Kentucky Fried Chicken for hospitality food during the focus group interviews. Indeed, other investigators have documented changes in the everyday diets of immigrant women toward more processed foods and animal proteins as well as foods high in fat, salt, or sugar [60, 61]. Evidence suggests that obesity is increasingly prevalent in post-migration women because of the adoption of a Western diet, likely due to “obesogenic” food environments in the vicinities of many immigrants’ residences [62]. Conversely, immigrant women may instead internalize the dominant body norms of their settlement home (i.e., the skinny model or actress common in media images in North America, Australia, and Europe) and reduce their dietary intake, even during reproduction, to maintain or quickly return to these hegemonic body ideals [63]. Cultural differences in food choices are exemplified in a comment made by a participant in our study:

Chinese or Asian basically, we don’t drink cold water after [birth], and they give you a bottle of ice chips … for us it’s totally unacceptable. And also, like cold foods – sandwich, salad, cereal. Oh, my gosh, we don’t eat those things. (IW- FGI-Urban Town)

For similar reasons, women from some ethnocultural groups are not comfortable bathing right after birth:

So right now when we have the prenatal class we always tell the clients, like if the ask you to go wash, take a bath, shampoo, whatever, just say yes and then you don’t need to do it. Don’t argue with them. (SSP-FGI-Urban Town)

Cultural clash was evident when the women shared their perspectives on caring for their newborns during the early postpartum period. In their countries of origin, most women are required to rest during the postnatal period while family members care for them and their new infant. In the Canadian context, IW may no longer have this support from their extended family while in hospital, and maternity care nurses do not extend this level of support.

My baby was crying during the night and I said [to nurse]: please can you take her in the nursery and so that she can be there and I can sleep … but she said: Here in North America, in Canada…you have to sleep with your baby. (IW-FGI- Urban Town)

Migrant women are reported to underutilize formal healthcare and other community services, largely because of language barriers, difficulties understanding healthcare information, experiences of discrimination, and challenges in navigating the Canadian healthcare system [24, 64, 65]. Moreover, the medicalized view of maternity promoted by Western biomedicine may powerfully influence immigrant women’s perceptions of maternal care in ways that may not be congruent with their frames of reference.

## Discussion

In light of the theories of organizational behaviour and critical realism, in this study barriers to accessing and navigating maternity care for immigrant women were explored at personal and structural levels. By studying these barriers in relation to organizational behaviour within healthcare units, we identified that although healthcare services are meant to be provided universally in the Canadian context, immigrant women experience inequity due to several personal and organizational barriers.

Maternity care in these situations fails to meet the specific needs of diverse populations and can be regarded as available but inaccessible to immigrant women of particular cultures. We also applied the theory of critical realism to understand the dialectical interplay between social structures (maternity care) and human agency (immigrant women) [34, 35]. Critical realism often addresses the causal relationship between personal (agency) and structural factors, such as norms, values, and beliefs that can influence an individual’s health and wellbeing [34, 37, 66]. Thus, to better understand the whole phenomenon and nature of the barriers and experiences, this study included three subpopulations: immigrant women, healthcare providers, and social service providers/stakeholders. This theoretical lens enabled us to elicit and uncover the multifaceted dimensions of barriers and experiences in respect of access to and navigation of maternity care services by immigrant women in rural and urban settings. Our findings indicate that some of the issues were commonly identified by all three study cohorts in both the rural and urban city sites in this study. Notably there is wide variation of demographic and ethnocultural profiles in Canadian cities. The critical realism theory illuminated our identification of the most critical points with respect to access to and navigation of maternity services by immigrant women. Some of these barriers are influenced by personal factors, such as lack of knowledge and low language proficiency by some of the immigrant women and discriminatory practices by some healthcare providers, while other factors are mostly structural, such as shortage of healthcare providers and inadequate professional interpreting services.

Inadequate language proficiency emerged as one of the main factors that limit access to Canadian maternity care services. This finding is supported by previous studies about immigrant health communication [15, 18, 67, 68]. A language barrier not only hinders communication but also limits access to the information resources about availability of services [15, 23]. Immigrant women who do not have information and knowledge about the availability of the services simply are not accessing these services. This challenges one important dimension of organizational behaviour theory, where the system aims to provide services universally, equally, and effectively for all members of the society [26]. As our findings revealed, although the services are available equally for all members of the society, in practice the services are not accessible to immigrant women due to lack of newcomer awareness about existence of these services. Consultation times were also considered problematic. Participants who thought the consultation times were insufficient suggested that times could be allocated selectively to reflect the purpose of the visit; for example, a routine visit could be shorter than a visit that involved a health concern. Physician attention during the consultation was another issue. Some participants noted that a physician’s attention during consultation was divided among conferring with the client, working with a computer, and writing notes – to the extent that the client had to request the physician’s attention. Participants felt that the physician was not interested in what they were saying if he or she was distracted by other activities.

There is evidence that besides language barriers, lack of social networks is associated with a lack of information [69]. Even though these sources of information may be informal, they form an important dimension. Some of the participants found that the information received contradicted their own customs and perceptions [25].

Our study supports previous findings that immigrant woman lose supportive networks from their home country and many do not find the social support they need in Canada [70, 71]. We found that lack of social support limits their ability to access maternity services, particularly when they have other children at home or no one to help them with transportation, as previously reported [70].

Another important issue related to organizational behaviour theory is that human resource challenges (healthcare provider shortage) can lead to negative or less satisfactory maternity outcomes [72]. Indeed, our findings revealed that a lack or shortage of healthcare providers impacts greatly on the issue of access to and navigation of maternity healthcare facilities by immigrant women, as some women in this study could not obtain a family doctor for almost a year. This finding is consistent with earlier studies addressing the difficulties of finding desired physicians in Canada [73, 74]. Timely access to healthcare services is a major aim of organizational behaviour theory; reductions in waiting times and in delays for receiving healthcare services are important for wellbeing [26]. A shortage of healthcare providers not only limits the reception of services in a timely manner, but also increases physician work load and may reduce consultation times, resulting in the provision of minimal services for all clients. In the consultation period, some of our participants perceived that that due to the physician’s workload, the physicians did not pay much attention to what they were saying.

A health organization needs to provide equal services for all clients, free of any stereotyping based on personal characteristics such as ethnicity or “race” [26, 74]. Our findings indicated that systematic [75] discrimination was experienced in Canada. In some situations, health insurance (Interim Federal Health) claims of refugees were not accepted by pharmacies and institutional discrimination was reported to be evident [18, 76]. This is mostly due to bureaucratic barriers that lead to the delay of payment by government and the time-consuming paper work required [18, 76, 77]. Other forms of discrimination were seen to be a result of lack of cultural competence [75]. Cultural competency helps to reduce disparities in healthcare organizations [31, 78]. Lack of awareness about “others’” cultural norms and values can lead to misunderstandings or stereotyping of populations. Findings from this study support the probability that some immigrant women may have origins in countries where health beliefs and behaviours are in opposition to Western medical beliefs. Many of the women continued to follow their healthcare beliefs and some experienced cultural shock when confronted with different healthcare practices such as the types of food served in maternity wards and less focused postnatal care. In a pluralistic multicultural society such as Canada, cultural competency of healthcare providers plays an important role in providing sufficient healthcare services with reduced risk of misunderstandings [23, 29].

Perceptions of inadequate care experiences by service users can lead to resistance to utilising Canadian maternity care and discourage IW from using services in future instances [15, 75].Wood and Newbold [15, 65] recommend having continuous cultural competency training of medical science students  and it is essential to achieve culturally competent health services to meet the needs of Canada’s multicultural society.

### Limitations and strengths

The study was conducted in one province in Canada, therefore our findings are not necessarily universally transferable to all contexts, globally. The inclusion of immigrant women, services providers, and other key stakeholders is a strength of the study, as a 360 degree perspective is obtained on a specified phenomenon. Whilst we adopted a multi-faceted recruitment strategy it is possible we may not have recruited women with very poor language fluency and immigrant women who experience the most marginalisation. Women who are recently arrived in a new ethno-culture because of pre-migration experiences (e.g. war, civil unrest and transgression of human rights) may be reluctant to criticize state institutions and formal health care services.

## Conclusions

Research on the reduction of barriers in accessing healthcare services is not only beneficial at personal and practical levels; it also supports important health policy and strategy messages. Clarity regarding the barriers, critical points, and other factors creating obstacles for immigrant women’s access to and navigation of healthcare services may help healthcare decision makers when planning and creating a more adequate system for meeting the needs of diverse societies.

Following immigration to a new ethnocultural context women may be faced with challenges such as diminished social networks and family support. Support from family and friends makes a difference because they can provide lay translation and interpretation services, accompany the women to healthcare appointments, provide child care during appointments, and encourage support during delivery.

We identify structural factors that limit access to maternity services by immigrant woman from various perspectives. The barriers to accessing maternity care were analysed using critical realism theory. Our findings indicate that maternity health services are not fully comprehensive and do not function at a level sufficient to serve immigrant women in the two cities in Alberta that received the study. Although data from qualitative research with a purposive sample obtained from two cities may not be generalized to the broader context of the country, many of the findings will be applicable to other jurisdictions, and to other countries. The results of this study show that maternity care in Canada would be hugely improved if the following recommendations were executed by health policy makers: (1) health informational packages need to be developed in different languages and dispersed widely throughout local populations; (2) continuous cultural competence programs, with a focus on personalized women- centered care, need to be created, and healthcare professionals must be required to undertake these programs; (3) healthcare professionals must be recruited from diverse ethnocultural groups; and (4) adequate monitoring and evaluation programs for prevention of personal and systemic discrimination must be implemented.
